# Four-dimensional multi-site photolysis of caged neurotransmitters

**DOI:** 10.3389/fncel.2013.00231

**Published:** 2013-12-02

**Authors:** Mary Ann Go, Minh-Son To, Christian Stricker, Stephen Redman, Hans-A. Bachor, Greg J. Stuart, Vincent R. Daria

**Affiliations:** ^1^Eccles Institute of Neuroscience, John Curtin School of Medical Research, The Australian National UniversityCanberra, ACT, Australia; ^2^Department of Human Physiology and Centre for Neuroscience, Flinders UniversityAdelaide, SA, Australia; ^3^Medical School, College of Medicine, Biology and Environment, The Australian National UniversityCanberra, ACT, Australia; ^4^Department of Quantum Science, Research School of Physics and Engineering, The Australian National UniversityCanberra, ACT, Australia

**Keywords:** two-photon photolysis, holographic projection, two-photon microscopy, caged neurotransmitters, synaptic integration

## Abstract

Neurons receive thousands of synaptic inputs that are distributed in space and time. The systematic study of how neurons process these inputs requires a technique to stimulate multiple yet highly targeted points of interest along the neuron's dendritic tree. Three-dimensional multi-focal patterns produced via holographic projection combined with two-photon photolysis of caged compounds can provide for highly localized release of neurotransmitters within each diffraction-limited focus, and in this way emulate simultaneous synaptic inputs to the neuron. However, this technique so far cannot achieve time-dependent stimulation patterns due to fundamental limitations of the hologram-encoding device and other factors that affect the consistency of controlled synaptic stimulation. Here, we report an advanced technique that enables the design and application of arbitrary spatio-temporal photostimulation patterns that resemble physiological synaptic inputs. By combining holographic projection with a programmable high-speed light-switching array, we have overcome temporal limitations with holographic projection, allowing us to mimic distributed activation of synaptic inputs leading to action potential generation. Our experiments uniquely demonstrate multi-site two-photon glutamate uncaging in three dimensions with submillisecond temporal resolution. Implementing this approach opens up new prospects for studying neuronal synaptic integration in four dimensions.

## 1. Introduction

Photostimulation using caged neurotransmitters (Callaway and Katz, 1993; Denk, 1994) and light-activated ion channels (Nagel et al., 2003; Boyden et al., 2005) have revolutionized the capacity to study the brain. Information processing in neuronal networks is achieved via signaling between neurons at synapses. Thus, controlled and localized activation of synapses offers the possibility to systematically study how individual neurons in the brain process information. Two-photon (2P) photolysis of caged compounds allows the simulation of highly localized release of neurotransmitter, enabling targeted activation of receptors at single synapses. Through activation of multiple synapses, it is possible to study how neurons integrate synaptic inputs to generate action potentials (APs). However, to date it has not been possible to activate synapses arbitrarily along the three-dimensional dendritic arbor of a neuron using realistic spatio-temporal [i.e., four-dimensional (4D)] patterns of light that resemble physiological inputs.

Laser-scanning systems utilizing galvanometer (GM) mirrors (Gasparini and Magee, 2001, Matsuzaki et al., 2001; Branco et al., 2010) implemented in conventional two-photon laser-scanning microscopes provide quasi-simultaneous multi-site stimulation by scanning the laser beam at fast rates (~10 kHz; i.e., switching time of ~100 μs). These scanning systems, however, are two-dimensional and are restricted to a single optical plane. A high-speed 3D scanning system has been described utilizing four acousto-optic modulators (AOMs) (Reddy et al., 2008). This system has been used to monitor neuronal activity via calcium imaging (Reddy et al., 2008; Katona et al., 2012). However, using four AOMs results in low optical throughput, and the dispersion through the AOM crystal significantly decreases 2P efficiency due to pulse broadening. Consequently, such a system has not been utilized for multi-site photolysis of caged neurotransmitters in 3D.

Beam-shaping techniques using a spatial light modulator (SLM) can split a single laser beam into several beamlets and generate arbitrary illumination patterns for photostimulation. These techniques have been demonstrated for photostimulation with shaped illumination in single-photon (1P) (Lutz et al., 2008; Zahid et al., 2010) and 2P (Dal Maschio et al., 2010; Papagiakoumou et al., 2010), and for a multi-foci photostimulation pattern in 1P (Anselmi et al., 2011; Yang et al., 2011) and 2P (Nikolenko et al., 2008). 1P excitation exhibits poor localized excitation along the optical axis. While 2P photostimulation offers the required axial resolution, previous studies have only showed stimulation in a single plane. We recently demonstrated 2P photolysis of caged neurotransmitters using holographic projection of an arbitrary 3D multi-foci uncaging pattern (Go et al., 2012). While holographic projection allows simultaneous multi-site photostimulation in 3D, the slow response of the SLM (~10–30 ms) remains the limiting factor in achieving fast switching light patterns within physiologically relevant timescales (~1 ms). Here, we overcome this limitation by temporally gating the 3D multi-foci uncaging pattern generated by the SLM using a high-speed spatial light switching array provided by a digital micro-mirror device (DMD). The programmable array of micro-mirrors can be used to independently control each beamlet in less than a millisecond (~0.7 ms) allowing the multi-site pattern to be changed with submillisecond speed. Random spatial stimulation patterns in 3D are therefore possible within physiologically relevant timescales. We demonstrate the performance of such a system as a tool for studying neuronal integration by uncaging glutamate at multiple foci with submillisecond resolution and thereby stimulate dendritic spines on different dendritic branches.

## 2. Materials and methods

### 2.1. Microscope design

**Figure 1:**
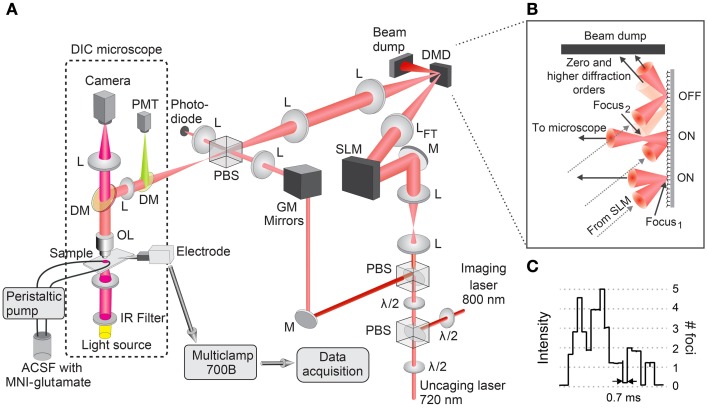
**Optical design of 4D holographic microscope. (A)** Light from a laser tuned to 800 nm is projected through two polarizing beam splitters (PBS) onto two galvanometer (GM) scanning mirrors that scan the excitation beam across the sample for imaging. Fluorescence from the sample is collected by a photomultiplier tube (PMT). Light from another laser tuned to 720 nm is expanded, projected onto a spatial light modulator (SLM), focused onto a digital micro-mirror device (DMD) and imaged onto the sample. The SLM is encoded with a phase hologram that generates a multi-focal excitation pattern from the incident laser beam. The DMD acts as a spatial light switch allowing independent switching of the individual foci. The resulting spatio-temporal excitation pattern at the sample plane is used for uncaging glutamate. λ/2, half-wave plate; DM, dicroic mirror; M, mirror; L, lens; L_*FT*_, Fourier transform lens; OL, objective lens; ACSF, artificial cerebro-spinal fluid. **(B)** The DMD switches the excitation focus ON and OFF by changing the tilt of the subset of programmable micro-mirrors in the corresponding focus (Focus_1_ if confocal with the objective lens). A larger subset of micro-mirrors is needed when the beam focus (Focus_2_) is off the DMD plane. In the ON state, the laser beam is directed to the objective lens; in the OFF state, to a beam dump. Zero and higher diffraction orders are also directed to the beam dump. **(C)** The number of foci turned ON is indicated by the laser intensity incident on the sample measured using the photodiode located in the conjugate Fourier plane. Minimum switching time with the DMD is 0.7 ms.

Our time-gated holographic microscope system employs an SLM, encoded with a phase hologram to generate a multi-focal excitation pattern from the incident laser beam, and a DMD, which acts as a spatial light switch allowing independent switching of the individual foci. The resulting spatio-temporal excitation pattern is used for glutamate uncaging. We also use GM scanning mirrors to image the 3D morphology of the neuron. A schematic of the system is described in Figure 1A. Two laser beams for imaging (800 nm) and uncaging (720 nm), respectively, are combined ahead of the objective lens via a polarizing beam splitter following re-orientation of the polarization of each laser using half-wave plates. GM scanning mirrors scan the linearly polarized imaging beam from a near infrared (NIR) Ti:S laser (Coherent Inc. MIRA 900) for 2P fluorescence imaging and a dichroic mirror reflects the beam to the objective. We obtain a 2P image by collecting the green fluorescence from the sample directed to a second dichroic mirror, which reflects wavelengths below 650 nm into a photomultiplier tube. The sample may also be viewed via an upright differential interference contrast (DIC) microscope (Olympus BX50WI). In DIC imaging mode, the dichroic mirror above the objective lens allows infrared light (>810 nm wavelength) to pass through and focus onto a charge-coupled device (CCD) camera (Dage-MTI IR-1000EX). The uncaging laser beam from a Ti:S laser (Coherent Inc. Chameleon) is expanded by a telescope to illuminate the 16 × 12 mm^2^ area of a programmable phase-only SLM (Hamamatsu X10468-02), where the phase-only hologram is encoded. The hologram is computed using custom software based on the standard prism-lens superposition algorithm (Liesener et al., 2000; Curtis et al., 2002). The DMD (DLP3000, Texas Instruments) is located at the Fourier plane with respect to the SLM and is interfaced to the microscope using 4*f* relay lenses, which position the DMD at the conjugate image plane with respect to the sample region. The DMD is used to individually switch each beamlet of the excitation pattern ON or OFF. In the ON state, the micro-mirrors direct the laser light to the sample (see Figure 1B). In the OFF state, the laser light is directed to a beam dump. The relay lenses are chosen to ensure that the back aperture of the objective lens is filled and that the area of the DMD encompasses the field of view of the objective lens (200 μm^2^ for a 1.0 NA 40× objective).

The dimension of one micro-mirror in the DMD is smaller than the diffraction-limited focal spot of the Fourier transform (FT) lens, with an effective numerical aperture (NA) of ~0.1. Hence, it takes a small set of micro-mirrors to switch a single beamlet of the multi-focal pattern ON and direct it to the sample for uncaging (see Figure 1B). When the holographic focus generated by the SLM is either above or below the plane of the DMD, a larger subset of micro-mirrors is needed. The size of the subset of micro-mirrors is calculated based on the conical angle of the focus with respect to the NA of the FT lens and the operating wavelength (720 nm). Figure 1C shows the relative intensity in the sample plane as measured by a photo-diode located at the conjugate Fourier plane (see Figure 1A) for different numbers of focal spots turned ON by the DMD. The intensity is more or less quantized and gives information on the number of foci active for uncaging. The DMD receives 24 bit RGB data at 60 Hz through its electronic video interface, with each color channel (RGB) having 8-bit depth. We operated the DMD in 1-bit monochrome mode. In this mode, a single image frame consists of 24 1-bit planes which encode the micro-mirror state at consecutive time epochs. Since each plane is weighted equally, this allows temporal sequences to be read out at 1440 Hz. By driving the DMD this way, the minimum switching time for a full ON–OFF cycle is 0.7 ms (see Figure 1C).

The setup is also equipped with a micromanipulator (Sutter Instruments), peristaltic pump (Gilson Minipuls 3) and amplifier (MultiClamp 700B, Molecular Devices) for electrophysiology. We use custom software developed in Labview (National Instruments) to control the acquisition of 3D 2P images, the calculation of the appropriate hologram for projection of photostimulation sites, and the laser intensity via a polarizing beam splitter and a half-wave plate on a motorized rotation mount.

### 2.2. Tissue preparation and electrophysiology

Three hundred micrometer thick slices of rat somatosensory cortex and hippocampus (P22–35) were prepared with a vibratome (Leica VT1200S). The slices were cut in ice-cold oxygenated artificial cerebrospinal fluid (ACSF) that contained (in mM): 1.25 NaH2PO4, 1.0 MgCl2, 125.0 NaCl, 2.5 KCl, 2.0 CaCl2, 25.0 NaHCO3, and 10.0 glucose. Slices were incubated in oxygenated ACSF at 34°C for 30 min and kept at room temperature before being transferred to the recording chamber. Animal experiments were performed with the protocol approved by the Animal Ethics Committee of the Australian National University.

We filled neurons through the recording patch pipette (*R* = 3.5–4.5 MΩ) with an intracellular solution containing (in mM): 115 K-gluconate, 20 KCl, 10 HEPES, 10 phospho-kreatine, 4 ATP-Mg, 0.3 GTP, 5.4 biocytin, and 0.1 Alexa-488 (Invitrogen). Whole-cell current clamp recordings of layer II/III and V pyramidal cells were obtained with a MultiClamp 700B. Current was injected when necessary to maintain a resting membrane potential of −70 mV. For the experiments in Figures 3D–F, the resting membrane potential was depolarized to −55 mV to allow for APs to be more easily generated. Data analysis was done with AxoGraph X and Matlab. We calculated peak currents and voltages by averaging 5–12 trials.

### 2.3. Two-photon imaging

Neurons filled with 0.1 mM Alexa-488 were imaged at 800 nm with 12–22 mW laser power. The dye was allowed to diffuse into the neuron for 20–30 min before imaging. Image stacks of 800 × 800 pixels in a single plane were generated by imaging individual planes in 1 μm increments along the *z*-axis. ImageJ (National Institute of Health) was used for 3D visualization.

### 2.4. Two-photon glutamate uncaging

We determined the potential sites on the dendritic tree for photostimulation from the 3D image map of the fluorescently labeled neuron. The appropriate phase hologram for the desired multi-focal pattern around the neuron was then calculated and encoded onto the SLM. The DMD was used to switch individual sites ON or OFF.

MNI-caged glutamate (Tocris Bioscience) was bath-applied (3 mM) and uncaged at 720 nm in the presence of 0.1 mM cyclothiazide using 9–30 mW power per uncaging spot. A closed recirculating system using a peristaltic pump was used to minimize the ACSF volume. An automated drift correction algorithm was run before every uncaging event to ensure that the photostimulation sites remained optimized.

## 3. Results

### 3.1. Time-gated holographic system

**Figure 2:**
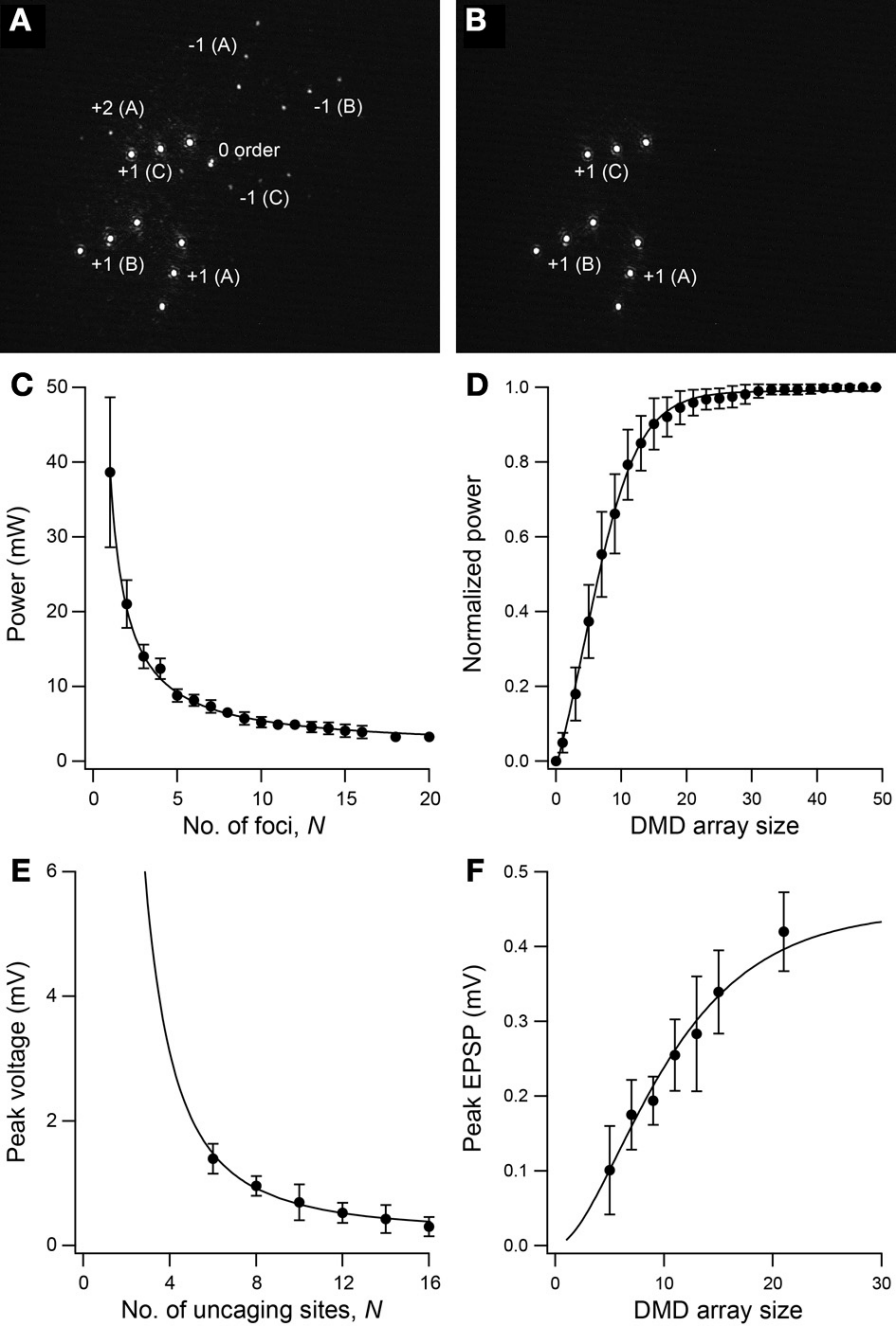
**SLM-generated 3D photostimulation pattern with DMD gating. (A)** Nine-foci light pattern produced by the SLM with all DMD pixels turned ON showing zero order (0 order), second order [+2 (A)] and mirror projections [−1 (A–C)] of the first diffraction order [+1 (A–C)]. **(B)** Same photostimulation pattern as in (A) but with only the nine desired foci gated by the DMD. All other light is eliminated by being directed to the beam dump. **(C)** Power on one ON focus as a function of number of foci, *N* (*n* = 10 different spot configurations). For *N* =2–20, the ON focus was kept at a fixed position. Solid line is 1/*N* fit. **(D)** Normalized power as a function of DMD array size (*n* = 10 focal spot positions). Solid line is a cumulative Gaussian fit. **(E,F)** Peak of glutamate-evoked voltage from one ON uncaging site as a function of **(E)** number of uncaging sites and **(F)** DMD array size (*n* = 8–10 trials). Solid lines are proportional to squares of curve fits in *C* and *D*, respectively.

Our microscope design builds on an earlier reported two-photon holographic microscope system capable of simultaneous multi-site photostimulation in 3D (Go et al., 2012). The incorporation of a DMD allows for full-cycle (OFF–ON–OFF) switching of photostimulation patterns within 0.7 ms. This is a rate that is not possible with just the SLM alone, which has a response time of ~10–30 ms. Although high-speed shutter systems can operate on the same submillisecond timescale, they do not allow independent switching of multiple foci. The addition of the DMD to our set-up also “cleans up” the 3D multi-site photostimulation pattern produced by the SLM. The DMD offers a convenient way to eliminate the spurious light patterns that normally result from holographic projection using a phase-only SLM with finite space-bandwidth product (Palima and Daria, 2006, 2007; Go et al., 2011). To visualize the different foci, we placed a reflective surface (e.g., glass slide) in the sample plane and imaged the reflected light leaked through the dichroic mirror directly above the objective lens (see Figure 1A). Figure 2A shows the photostimulation pattern produced by the SLM projected onto the sample with all DMD pixels in the ON position. Figure 2B shows the same photostimulation pattern with only the intended nine foci switched ON. Note that the undiffracted (i.e., zero-order; see image center in Figure 2A) beam from the SLM is missing in the latter, as are other higher diffraction orders and mirror projections of the first orders. The DMD therefore provides an easy way of eliminating the zero-order beam, thus, eliminating the risk of unintended uncaging, and gives independent control for switching each stimulation site.

Figure 2C shows how the power on one uncaging focus depends on the number of focal spots, *N*. The relationship is an inverse proportionality as reported earlier (Daria et al., 2009). The error bar for *N* = 1 shows the variability in power among different non-central positions of the holographic spot which results from the spatial variation in the diffraction efficiency of the SLM. For *N* = 2 to 20, the ON focus was kept at a fixed position as the spot configuration for a fixed number of foci was varied. Note that the variability in power within a single position is much less than the spatial variability. The laser beam has a Gaussian intensity profile. Figure 2D shows the normalized power as a cumulative Gaussian function of DMD array size, which defines the length of the square array of micro-mirrors for gating the uncaging focus, averaged over different positions of the holographic spot. On average, 96% of the maximum intensity at each spot was recovered using a DMD array size of 21, whereas 90% was recovered with an array size of 15. Based on this information, for most of the experiments in this paper we used an array size of 15. Figures 2E,F show how the glutamate uncaging-evoked response varies with number of uncaging sites, *N*, and DMD array size, respectively. The quadratic intensity dependence of 2P absorption is reflected here with the peak voltage seen as a function of 1/*N*^2^ and a squared cumulative Gaussian function of DMD array size. As the total laser power was kept constant for the experiment in Figure 2E, data points for *N*<6 were not taken to preclude damaging the DMD with high laser power. Laser pulse duration for Figures 2E,F is 2 ms.

**Figure 3:**
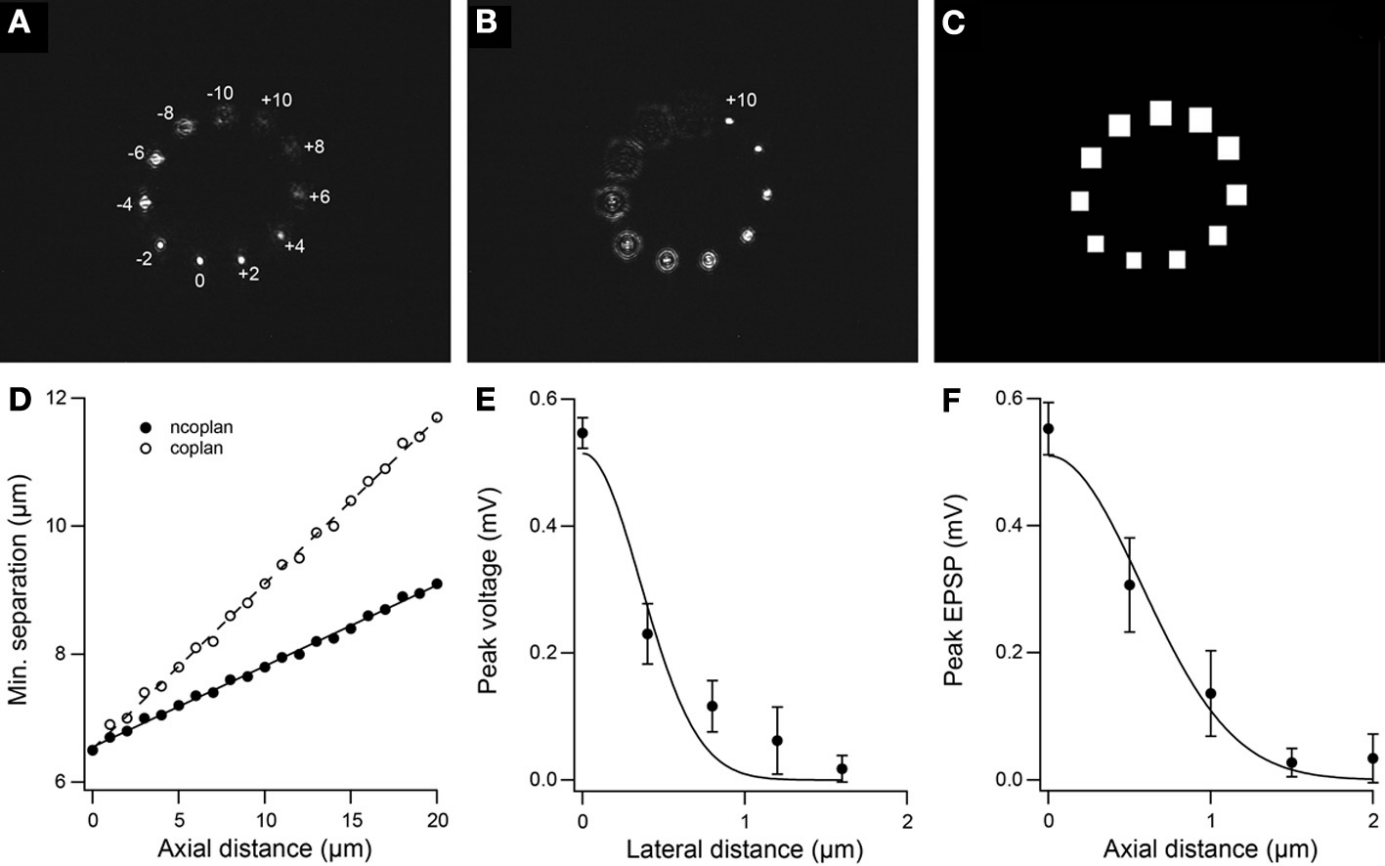
**Spatial resolution of independent gating and uncaging. (A)** 3D photostimulation pattern with objective lens focused in the reference plane *z* = 0 μm and the other 10 sites holographically projected either above (negative) or below (positive) the reference plane. **(B)** Same photostimulation pattern as in *C* but with objective lens focused in the plane *z* = +10 μm. **(C)** Sizes of micro-mirror arrays for switching foci shown in *A*. The array size scales with distance from the objective focal plane allowing collection of out-of-focus light. **(D)** Minimum separation for independent gating of two uncaging sites as a function of axial distance for the case when one spot is fixed while the other spot is moved away axially (ncoplan) and for when both spots move together in the axial direction (coplan). DMD array size is 15. Solid line is linear fit. **(E)** Peak voltage with varying lateral distance orthogonal to a spine. FWHM =0.8 ± 0.1 μm. **(F)** Peak voltage as a function of axial distance above the spine. FWHM = 1.3 ± 0.1 μm. For *E* and *F*, solid lines are Gaussian fits.

Figure 3A shows 11 foci positioned in a circular arrangement with *z*-axis positions ranging from 10 μm above (negative) to 10 μm below (positive) the nominal focus of the objective lens (0 μm). The point in the focal plane is in focus (i.e., sharpest) but the rest are out of focus with the points in the farthest away planes (±10 μm) the most out of focus. Figure 3B shows the same light pattern but with the objective lens focused in the plane *z* = +10 μm. Figure 3C shows the DMD array sizes for gating the different foci in the light pattern in Figure 3A. By scaling the micro-miror array size for gating the relevant site with distance from the nominal focal plane of the objective lens, we accommodate the increasing size of the out-of-focus projection of the beamlets and allow collection of light for points projected above and below the focal plane. The DMD array size puts a restriction on the minimum separation between two uncaging sites for independent gating. Figure 3D plots the minimum distance between two uncaging sites as a function of axial distance for an array size of 15 for the case where one spot is fixed while the other spot is moved away axially and for when both spots move together in the axial direction. The deviation of data points from a straight line is due to the DMD having a finite number of discrete pixels. The minimum separation for two uncaging sites in the same plane is 6.5 μm. We refrained from positioning uncaging sites within 10 μm of the center to avoid switching ON the zero-order beam and unnecessarily illuminating the sample. Technically, however, this minimum separation is a soft limit as independent uncaging at two sites, defined by the spatial resolution of glutamate uncaging, has a much narrower profile. Figures 3E,F show representative lateral and axial resolution profiles from uncaging-evoked voltage responses to 700 μs laser pulse duration. The responses were measured in current clamp as the uncaging site was moved orthogonally from the spine while the objective was kept fixed. The lateral profile has a full width at half maximum (FWHM) of 0.8 ± 0.1 μm. The axial profile was 1.3 ± 0.1 μm. The solid lines correspond to Gaussian curve fits. The final resolution depend on laser power, laser pulse duration and local MNI-glutamate concentration. For example, we earlier reported uncaging resolution with FWHM of 2.4 ± 0.2 μm and 3.7 ± 0.3 μm for the lateral and axial profiles, respectively, using 2 ms laser pulse duration (Go et al., 2012).

### 3.2. Spatio-temporal patterned stimuli

**Figure 4:**
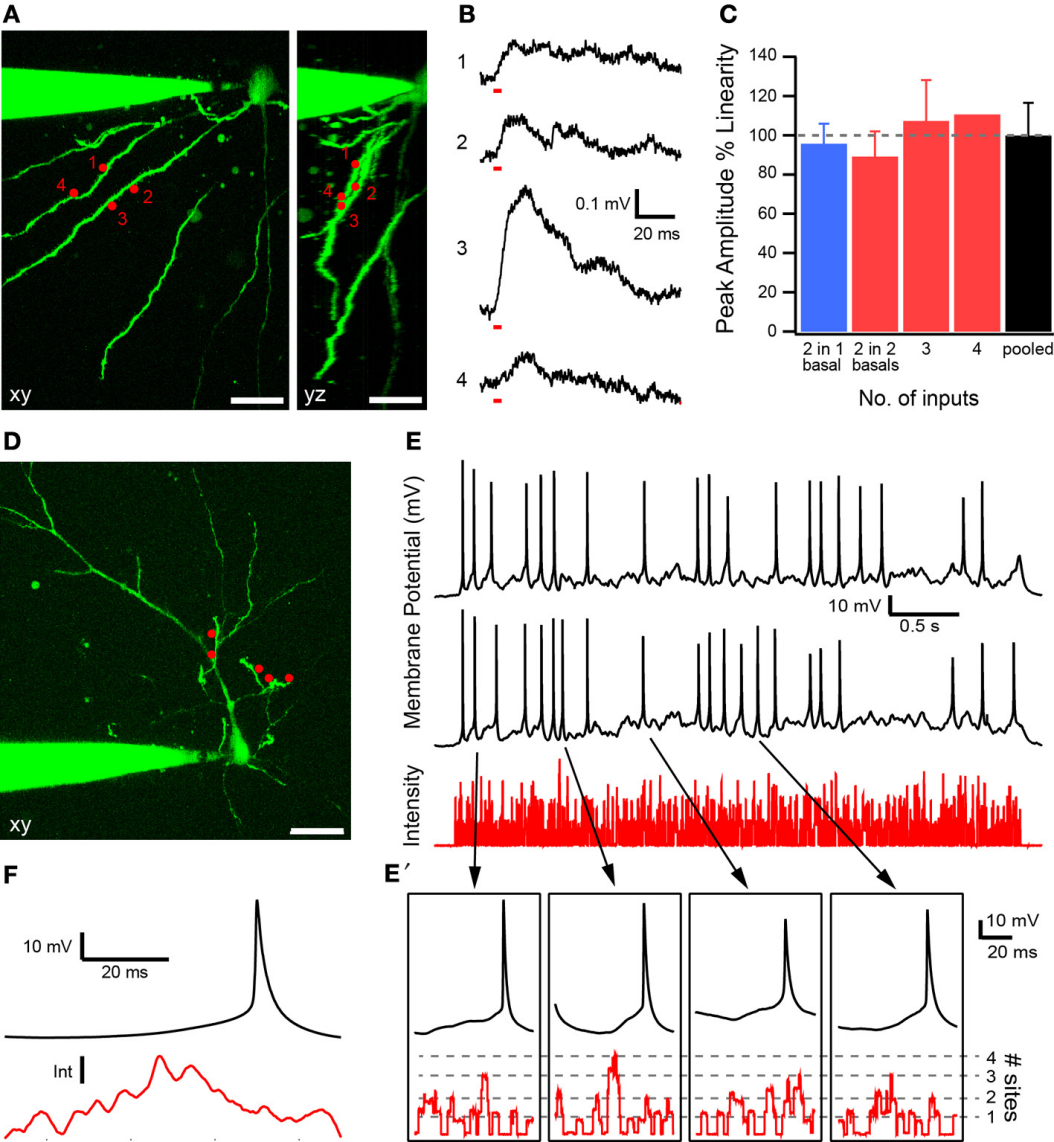
**Synaptic integration experiments. (A)** Layer V pyramidal cell with uncaging sites in two planes 5 μm apart (1 and 2 are in one plane, 3 and 4 in another) as viewed in the *xy* and *yz* planes. Scale bar 25 μm. **(B)** Individual uncaging-evoked EPSPs obtained by turning ON stimulation sites in a static hologram one at a time with the DMD. Stimulus duration (3 ms) is indicated by red bars at the start of the EPSP. **(C)** Summary of results for different combinations of uncaging sites. Linearity is expressed as the ratio of the peak amplitudes of the measured compound EPSP and the arithmetic sum of individual EPSPs. Dashed line denotes linear summation (100%). Blue bar corresponds to within-branch summation; red, between-branch summation. Data are shown as mean ± s.e.m. for all combinations. **(D)** Layer II/III pyramidal cell with five uncaging sites situated along three apical oblique branches and all in a single plane. Scale bar 25 μm. **(E)** Representative membrane potential time courses generated by two repeats of the same train of random stimulus for uncaging glutamate at the sites in *D*. In the stimulus, the spatial selections and their time intervals are both random. The resting membrane potential at the soma was set to −55 mV. Several action potentials (APs) align in time triggered by the same uncaging patterns. Laser intensity in the sample plane is indicative of the number of simultaneously active stimulus sites. **(E')** Magnified view showing membrane potential (black) and laser intensity (red) for a time window from 60 ms before to 20 ms after the peak of the AP. **(F)** Mean membrane potential and laser intensity (*n* = 53 APs) from 60 ms preceding peak of AP to 20 ms after. AP is driven by a significant increase in laser intensity shortly (~23 ms) before its peak.

The system offers access to the 3D dendritic arbor of the neuron for activating synaptic receptors. This enables the study of how neurons integrate multiple synaptic inputs on their dendritic trees. We illustrate such an experiment in Figure 4. A 2P fluorescence image of a layer V pyramidal neuron is shown in Figure 4A with the recording pipette at the soma and some basal dendrites either viewed from the top (*xy*) or side (*yz*). Four uncaging sites are chosen with two each projected onto two basal dendrites located 5 μm apart in the vertical direction. A static hologram for all four sites is generated with the SLM, and the DMD is used to switch ON one focus at a time. Figure 4B shows glutamate-evoked excitatory postsynaptic potentials (EPSPs) generated by individually uncaging glutamate at each site for a duration of 3 ms. Laser power was set to generate EPSPs consistent with single synaptic inputs (<0.5 mV) (Nevian et al., 2007; Fino et al., 2009). Figure 4C summarizes EPSP summation for all the possible combinations of the uncaging sites. Linearity is expressed as the ratio of the peak amplitude of the composite EPSP generated when all sites are simultaneously stimulated and the arithmetic sum of individual EPSPs. The blue bar corresponds to within-branch and the red to between-branch summation, respectively. No statistically significant difference is observed, indicating that the summation is largely linear (two inputs in one basal, *n* = 2; two inputs on two basal dendrites, *n* = 4; three inputs, *n* = 4; four inputs, *n* = 1; pooled data, *n* = 11 combinations). This observed linear within-branch summation is consistent with the observation by Polsky et al. (2004) who showed that two weak EPSPs on the same layer V basal dendrite 20 μm apart summate linearly.

The DMD, by enabling independent switching of individual photostimulation sites, makes it possible to activate different combinations of the sites without having to change the phase hologram; i.e., the hologram remains static and sites are switched ON/OFF by the DMD. Moreover, as different stimulus combinations need to be compared, it is critical that the power at each uncaging site is kept constant. The DMD allows this and eliminates the necessity to adjust the total power for distribution among the uncaging sites when using different numbers of stimulus sites in this experiment. In addition, because the hologram is kept static and the power at each site constant, the power variability due to the spatially varying diffraction efficiency of the SLM is not an issue.

Next, we explored if fast multi-site photostimulation patterns could elicit APs. Figure 4D shows five marked uncaging sites on three apical oblique dendrites of a layer II/III pyramidal neuron. The DMD is used to randomly switch uncaging sites ON or OFF for 0.7–4 ms at a time for a total period of 4 s. Figure 4E shows the relative intensity at the sample, indicating the number of stimulus sites at which neurotransmitter is released due to uncaging, together with the membrane potential at the soma for two trials of identical time-varying uncaging patterns. Several peaks of the APs during the two trials align in time indicating that the APs are not randomly evoked, but are triggered by specific uncaging patterns. Figure 4E' shows a magnified view of sections of the membrane potential trajectory and laser intensity 60 ms before to 20 ms after the peak for four of the APs generated by this uncaging pattern. Integration of several inputs is required prior to AP generation. Figure 4F shows the average membrane potential and laser intensity (*n* = 53 APs) from 60 ms preceding peak of the AP to 20 ms after. On average, an AP is driven by a significant increase in laser intensity ~23 ms before its peak.

## 4. Discussion

### 4.1. Applications of a time-gated holographic system in neuroscience

We have shown that holographic projection with high-speed temporal gating via a DMD overcomes the 2D limitation of GM-based laser scanning systems and the poor temporal resolution of SLM-based holographic projection systems for 2P uncaging of neurotransmitters. The DMD allows independent control of each focus in the SLM-generated photostimulation pattern and offers a convenient way to eliminate the zero and higher diffraction orders. This method offers unprecedented flexibility in the design of spatio-temporal (or 4D) light patterns for highly localized release of neurotransmitters. It opens up a fresh possibility to study synaptic integration in 3D with high temporal resolution. Since holographic projection allows easy positioning of photostimulation sites along the dendritic arbor of neurons, this method allows integration between branches to be directly investigated. This is crucial since in pyramidal neurons, which are abundant in several regions of the central nervous system in mammals (Spruston, 2008), apical oblique and basal dendrites collectively receive the vast majority of excitatory synapses (Larkman, 1991; Megias et al., 2001; Wang et al., 2007). This method could also be applied to the spatio-temporal control of neuronal activity which allows probing of neural coding, i.e., what kinds of spatio-temporal input patterns a neuron responds to. In addition, uncaging may be extended to neuronal populations. By generating a multi-cell holographic stimulation pattern and using the DMD to sequentially evoke suprathreshold 2P activation of neurons in a population, connections to a patched neuron may be determined. This facilitates a fast method for finding connected pairs, for instance. This multiplexing technique may also be used for population calcium imaging in the study of network activity (Ducros et al., 2013) and the interaction between different subnetworks. Another potential field of application for these 4D light patterns is optogenetics. By using sculptured light to activate specific subnetworks of genetically labeled neuronal populations, an even higher degree of selectivity can be achieved than is possible with traditional optogenetic approaches alone (Liu et al., 2012; Lovett-Barron et al., 2012).

### 4.2. Relationship with other work

The basic component of holographic projection is a phase-only liquid crystal (LC) SLM encoded with a computer-generated phase hologram. The response time of the LC is dependent on its phase retardation rate, which relates to the operating wavelength of the SLM (Wu, 1986). This response time is typically ~10 ms, but addressing via computer video output further limits the temporal resolution to ~30 ms. There have been several attempts to increase the response time of SLMs (Dayton et al., 2001; Kirby and Love, 2004), but no technique using spatially addressable SLMs has achieved multi-level phase-only modulation to perform a full OFF–ON–OFF cycle of the hologram at submillisecond timescales. Thalhammer et al. (2013) recently demonstrated high-speed hologram transition using a device with an extended phase map of up to 4π and choosing the minimum route in phase shift for each pixel in between transitions. This technique allows for one-way transitions of 1–3 ms. An iterative hologram optimization routine is necessary to improve the diffraction efficiency with the reduced phase representation of the hologram. While this technique can potentially be applied to 4D photostimulation, changing the hologram introduces spurious light patterns during hologram transitions, which could result in unwanted uncaging. While a high-speed shutter or an acousto-optic deflector can be used to turn OFF the light during transitions, the projection of zero and higher diffraction orders can still pose a potential issue when the hologram is ON especially when the iterative optimization routine does not produce an efficient hologram for a specific stimulation pattern.

Holographic projection with binary phase modulations (0 and π) has been demonstrated recently for the study of neuronal circuits (Reutsky-Gefen et al., 2013). SLMs employing ferroelectric LCs have fast response times (~0.5 ms). However, binary holograms produce mirror projections of equal light intensity in addition to the zero order. This reduces the power available for stimulation (after blocking zero order and mirror projection) to significantly less than 40%. While such technique can well be applied for 1P excitation, either for optogenetics or uncaging, its low optical throughput can be an issue when applying the technique to 2P uncaging in 4D. Moreover, even for a fixed number of foci, global changes in the binary hologram impact the intensity level of each focus and could introduce variable 2P uncaging responses. Changing the number of foci necessitates dynamic control of the laser intensity affecting both 1P and 2P uncaging modes.

Our technique uniquely distinguishes itself from existing techniques in that it uses a conventional phase-only SLM with slow refresh rate combined with a DMD, which enables independent gating of the holographically projected multiple foci at high speeds. Here, the SLM first modulates the phase of the incoming laser beam to generate the desired multi-focal spatial profile before the DMD allows each focus to be individually turned ON or OFF. Thus, there is minimal loss of laser intensity compared to the method of using DMDs to remove unwanted light from a wide-field illumination to shape the excitation light (Bednarkiewicz et al., 2008; Zhu et al., 2012).

### 4.3. Challenges and future developments

The DMD array size sets a minimum lateral distance between two uncaging sites for independent gating. For an array size of 15, the minimum distance is 6.5 μm. This is a soft limit as glutamate uncaging has a much narrower profile (0.8 ± 0.1 μm lateral FWHM) and considerable overlap between two DMD arrays is needed to induce two-photon uncaging in the OFF site. However, we can further decrease this distance with a slight adjustment in the optical system. By replacing the FT lens (see L_*FT*_ in Figure 1A) between the SLM and DMD with one with a shorter focal length, we can achieve smaller foci on the DMD and consequently require smaller DMD array sizes for gating. This, however, will increase the incident power density level on the DMD and will require high-tolerance mirror coatings to prevent damage.

Our system uses a DMD (DLP3000) from an evaluation module (DLP LightCrafter, Texas Instruments). We retained only the components in the module necessary to address the bare DMD via the video interface and removed the other components (e.g., light source and projection lenses). The DMD is electronically driven at 1440 Hz when delivering the uncaging binary pattern via the computer's digital video output. This rate can be increased up to 4 kHz (250 μs) when 96 frames are preloaded onto the on-board memory of the DMD's driver circuit but this would give a total of only 24 ms of stimulation. Alternatively, one can use more advanced DMD systems (e.g., DLP V-module, ViALUX), which are capable of switching rates of up to 22 kHz. One limitation in this work is the low damage threshold of the DMD component itself. Since it is not designed for use with high power light sources (e.g., lasers), illuminating the DMD for long periods with focused laser spots damages the device. Such a technical constraint could be overcome by custom fabricating DMDs with specific mirror coatings to tolerate higher power levels. In this work, the DMD was potentially stretched to its limits by positioning it in the Fourier plane where the holographically projected foci from the 2P laser were incident. Long sequences of spatio-temporal stimulation patterns are not possible with the current device. Nonetheless, even with the limits of the current device it is apparent that the combination of SLM and DMD can be cooperatively applied to the study of synaptic integration.

In summary, holographic projection using an SLM combined with high-speed temporal gating via a DMD allows the generation of random spatio-temporal stimulation patterns in 3D with submillisecond temporal resolution, and in this way offers unprecedented flexibility in the design of 4D light patterns for highly localized release of neurotransmitters. Implementing this approach opens up a wide range of prospects for the study of neuronal circuits.

## Conflict of interest statement

The authors declare that the research was conducted in the absence of any commercial or financial relationships that could be construed as a potential conflict of interest.

## Supplementary material

**Supplementary movie file showing DMD switching of SLM-generated multi-focal photostimulation pattern.**
